# Genome-wide analysis of the *Populus Hsp90* gene family reveals differential expression patterns, localization, and heat stress responses

**DOI:** 10.1186/1471-2164-14-532

**Published:** 2013-08-05

**Authors:** Jin Zhang, Jianbo Li, Bobin Liu, Li Zhang, Jun Chen, Mengzhu Lu

**Affiliations:** 1 State Key Laboratory of Tree Genetics and Breeding, Research Institute of Forestry, Chinese Academy of Forestry, Beijing 100091, China

**Keywords:** Expression analysis, Gene family, Gene structure, Hsp90, Phylogenetic analysis, *Populus*

## Abstract

**Background:**

Members of the heat shock protein 90 (Hsp90) class of proteins are evolutionarily conserved molecular chaperones. They are involved in protein folding, assembly, stabilization, activation, and degradation in many normal cellular processes and under stress conditions. Unlike many other well-characterized molecular chaperones, Hsp90s play key roles in signal transduction, cell-cycle control, genomic silencing, and protein trafficking. However, no systematic analysis of genome organization, gene structure, and expression compendium has been performed in the *Populus* model tree genus to date.

**Results:**

We performed a comprehensive analysis of the *Populus Hsp90* gene family and identified 10 *Populus Hsp90* genes, which were phylogenetically clustered into two major groups. Gene structure and motif composition are relatively conserved in each group. In *Populus trichocarpa*, we identified three paralogous pairs, among which the *PtHsp90-5a/PtHsp90-5b* paralogous pair might be created by duplication of a genome segment. Subcellular localization analysis shows that PtHsp90 members are localized in different subcellular compartments. PtHsp90-3 is localized both in the nucleus and in the cytoplasm, PtHsp90-5a and PtHsp90-5b are in chloroplasts, and PtHsp90-7 is in the endoplasmic reticulum (ER). Furthermore, microarray and semi-quantitative real-time RT-PCR analyses show that a number of *Populus Hsp90* genes are differentially expressed upon exposure to various stresses.

**Conclusions:**

The gene structure and motif composition of *PtHsp90s* are highly conserved among group members, suggesting that members of the same group may also have conserved functions. Microarray and RT-PCR analyses show that most *PtHsp90s* were induced by various stresses, including heat stress. Collectively, these observations lay the foundation for future efforts to unravel the biological roles of *PtHsp90* genes.

## Background

Plants are exposed to various environmental stresses. Primary stresses such as high light intensity, heat shock, drought, chilling, salinity, and chemical pollutants act simultaneously on plants, causing cell injury and producing secondary stresses such as osmotic and oxidative stresses [1]. Plants cannot avoid exposure to these factors, but adapted morphologically and physiologically by some mechanisms. Biosynthesis of many proteins called “stress proteins” is induced to protect cells from these harmful stimuli [2].

Heat shock proteins (Hsps) are responsible for protein folding, assembly, translocation, and degradation in many normal cellular processes. They stabilize proteins and membranes, and can assist in protein refolding under stress conditions. They also play a crucial role in protecting plants from stresses by reestablishing normal protein conformations and thus cellular homeostasis [1]. Plant Hsps are classified into five families according to their molecular size: Hsp100, Hsp90, Hsp70, Hsp60, and small Hsps (sHsps). They have been well characterized in a few model plants such as the tomato, *Arabidopsis*, and rice [3,4].

Hsp90s are a class of chaperone proteins that are highly conserved in prokaryotes and all eukaryotes. They are the major species of molecular chaperones and require ATP for their functions [1]. Although *Hsp90s* are expressed in most organisms, their expression increases in response to stresses. Distinct from many other well-characterized molecular chaperones, Hsp90s display considerable specificity for their client proteins. Most of their known substrates are signal-transduction proteins such as steroid hormone receptors and signaling kinases [5]. Although the major function of Hsp90s is to assist protein folding, they play key roles in signal transduction, cell-cycle control, protein degradation, genomic silencing, and protein trafficking [5,6]. Expression of *Hsp90* in *Arabidopsis* is developmentally regulated and is responsive to heat, cold, salinity, heavy metals, phytohormones, and light and dark transitions [4,7]. Tobacco NbHsp90-1 and *Arabidopsis* AtHsp90-2 confer pathogen resistance by reacting to resistance proteins (R proteins), which are signal receptors from the pathogen [8,9]. In addition, Hsp90s interact with the 26S proteasome and play a key role in its ATP-dependent assembly and maintenance in budding yeast [10]. To fulfill their cellular roles, Hsp90s cooperate with other chaperones to form a multiprotein chaperone complex [11]. Moreover, Hsp90s also act as buffers to phenotypic changes and are portrayed as “capacitors for evolution” [12].

Hsp90s are encoded by multiple genes. They consist of conserved N-terminal and C-terminal domains that are joined by a charged linker region that varies in length. Genes encoding cytosol-, ER-, and plastid-localized Hsp90 proteins have been characterized in several plant species [4]. In the *Arabidopsis* genome, seven *Hsp90* family members have been identified. Sequence analyses of *Arabidopsis Hsp90* family genes have revealed two major subfamilies. AtHsp90-1–4 proteins containing the C-terminal pentapeptide MEEVD form the cytoplasmic subfamily; AtHsp90-5–7 form the other subfamily. AtHsp90-5 and AtHsp90-7 are localized in chloroplasts [13] and the endoplasmic reticulum (ER) [14], respectively. AtHsp90-6 is localized in mitochondria [15]. Overexpression of cytosolic AtHsp90-2, chloroplast-localized AtHsp90-5, and ER-localized AtHsp90-7 reduces tolerance to salt and drought stresses, but improves tolerance to high concentrations of Ca^2+^[16]. The induction of ABA-responsive genes is delayed by overexpression of cytosolic AtHsp90-2, but is hardly affected by overexpression of AtHsp90-5 and AtHsp90-7 under conditions of salt and drought stress, which implies that different cellular compartment-localized Hsp90s in *Arabidopsis* might contribute to responses to abiotic stresses by different functional mechanisms, probably through ABA- or Ca^2+^-dependent pathways [16].

The *Populus* genus comprises woody plants that are important to humans and animals. Completion of the *P*. *trichocarpa* genome sequence in 2006 rendered it a model species for research on trees [17], providing an opportunity to analyze and further understand Hsp90s. To determine the structure-function relationship of Hsp90s in the *Populus* genus, we performed detailed systematic analyses of genome organization, gene structure, and expression compendium. We report the comprehensive genomic identification and phylogenetic analysis of all 10 members of the *Hsp90* gene family in the *Populus* genus, as well as their expression profiles in different tissues and their responses under heat stress. Our results provide a framework for further functional investigations of these genes.

## Results and discussion

### Identification of the *Hsp90* gene family in *P*. *trichocarpa* and other plant species

To identify putative *Populus Hsp90* genes, we first searched relevant databases using the corresponding *Arabidopsis* Hsp90 protein sequences as queries. Additional searches were performed based on keyword querying. After removing redundant sequences, we identified 10 candidate *Hsp90* sequences in the genome of *P*. *trichocarpa*. All *PtHsp90* candidates were analyzed using the Conserved Domain Database (CCD) [18,19] and Pfam (http://pfam.sanger.ac.uk/). It was previously reported that there are seven *Hsp90* genes presented in *Arabidopsis*[4]. The number of *Hsp90* genes in *P. trichocarpa* genome is in consistency with the ratio of 1.4-1.6 putative poplar homologs for each *Arabidopsis* gene according to comparative genomics studies [17]. This indicates that the higher number of Hsp90 members in poplar is due to the expansion of gene families during the genome duplication and the genomic evolution followed. The *Hsp90* genes identified in *P*. *trichocarpa* encode proteins ranging from 698 to 823 amino acids (aa) in length, with predicted isoelectric points (pIs) ranging from 4.85 to 5.53 (Table 1). The polypeptides are also predicted to contain a Histidine kinase-like ATPases (HATPase_c) family motif and a Hsp90 family motif (Additional file 1). HATPase_c domain belongs to the ATP binding superfamily including diverse protein families such as DNA topoisomerase II, molecular chaperones Hsp90, DNA-mismatch-repair enzymes, phytochrome-like ATPases and histidine kinases [20]. Detailed information on the *Hsp90* family genes in *P*. *trichocarpa*, *Arabidopsis*, and rice is given in Table 1 and Additional file 1.

**Table 1:** **Hsp90 genes families in *Arabidopsis*, *Populus*, and rice**

**Gene name**	**Locus**	**Genomic position**	**Mol. Wt. (kDa), Length (aa), pI**	**Gene length, ORF, introns**	POSRT predictions*
*AtHsp90-1*	At5g52640	Chr5:21352542-21355147 (+)	81.18, 705, 4.95	2606, 2118, 3	N: 6, C: 5, Ch: 2
*AtHsp90-2*	At5g56030	Chr5:22686923-22689433 (+)	80.06, 699, 4.95	2511, 2187, 2	C: 11, P: 2
*AtHsp90-3*	At5g56010	Chr5:22681410-22683911 (+)	80.05, 699, 4.95	2502, 2100, 2	C: 9, P: 2, N: 1, M: 1
*AtHsp90-4*	At5g56000	Chr5:22677602-22680067 (-)	80.14, 699, 4.96	2466, 2100, 2	C: 9, P: 4
*AtHsp90-5*	At2g04030	Chr2:1281983-1285909 (+)	88.66, 780, 4.93	3927, 2343, 18	Ch: 12, N: 1
*AtHsp90-6*	At3g07770	Chr3:2479611-2483970 (+)	90.57, 799, 5.26	4360, 2400, 19	M: 9.5, Ch_M: 6.5, Ch: 2.5
*AtHsp90-7*	At4g24190	Chr4:12551902-12555851 (-)	94.2, 823, 4.94	3950, 2472, 14	E.R.: 7, V: 4, N: 1
*PtHsp90-1a*	Potri.004G073600	Chr04: 6143799 - 6146941(-)	80.72, 703, 5	2566, 2112, 3	C: 7, N: 3, P: 2, M: 1
*PtHsp90-1b*	Potri.017G146600	Chr17: 15369800 - 15373207(+)	80.75, 703, 4.98	2696, 2112, 3	C: 8, N: 2, P: 2, M: 1
*PtHsp90-2*	Potri.001G466000	Chr01: 49936276 - 49939430(+)	80.03, 699, 4.95	2767, 2100, 2	C: 6, P: 2, E.R.: 2, Ch: 1, N: 1, M: 1
*PtHsp90-3*	Potri.016G003400	Chr16: 167702 - 171110(+)	79.95, 698, 4.94	2906, 2097, 3	N: 7, C: 5, Ch: 2
*PtHsp90-4a*	Potri.001G286700	Chr01: 29295547 - 29298729(+)	80.86, 706, 4.88	2734, 2121, 2	C: 7, N: 2, P: 2, Ch: 1, M: 1
*PtHsp90-4b*	Potri.006G002800	Chr06: 219117 - 222439(+)	80.01, 699, 4.91	2734, 2100, 2	N: 4, C: 4, P: 3, Ch: 1, V: 1
*PtHsp90-5a*	Potri.008G112700	Chr08: 7190800 - 7197203(+)	90.16, 791, 4.92	5884, 2376, 17	Ch: 10, N: 2, P: 1
*PtHsp90-5b*	Potri.010G136800	Chr10: 15004039 - 15010595(-)	90.15, 793, 4.96	5723, 2382, 17	Ch: 13
*PtHsp90-6*	Potri.014G164900	Chr14: 13043761 - 13049852(-)	82.95, 723, 5.53	4850, 2172, 13	M: 7.5, Ch_M: 7, Ch: 5.5
*PtHsp90-7*	Potri.005G241100	Chr05: 24712685 - 24717968(-)	94.05, 823, 4.85	4926, 2472, 14	E.R.: 5, V: 5, P: 2, Ch: 1
*OsHsp90-1*	LOC_Os04g01740	Chr4: 483241 - 486065 (+)	80.25, 703, 5.04	2353, 2112, 2	C: 11, Ch: 1, P: 1
*OsHsp90-2*	LOC_Os08g39140	Chr8: 24719086 - 24723553 (-)	80.19, 699, 4.99	4002, 2100, 2	C: 8, P: 2, Ch: 1, N: 1, M: 1
*OsHsp90-3*	LOC_Os09g30412	Chr9: 18514572 - 18518316 (-)	80.2, 699, 4.97	3232, 2100, 2	N: 7, C: 4, Ch: 2
*OsHsp90-4*	LOC_Os09g30418	Chr9: 18535746 - 18541109 (-)	94.2, 830, 5.15	5364, 2493, 3	N: 8, C: 3, Ch: 1, P: 1
*OsHsp90-5a*	LOC_Os08g38086	Chr8: 24124838 - 24129488 (-)	85.85, 761, 4.97	4285, 2286, 19	Ch: 12.5, Ch_M: 7
*OsHsp90-5b*	LOC_Os09g29840	Chr9: 18150618 - 18155512 (-)	89.22, 791, 5.03	4359, 2376, 18	Ch: 12.5, Ch_M: 7
*OsHsp90-6*	LOC_Os12g32986	Chr12: 19921576 - 19927766 (-)	91.48, 811, 5.21	5680, 2436, 19	Ch: 12, M: 1
*OsHsp90-7*	LOC_Os06g50300	Chr6: 30444411 - 30450497 (-)	93.04, 812, 4.89	5669, 2439, 14	E.R.: 5, V: 5, P: 2, Ch: 1

Gene loci are obtained from the Phytozome website (http://www.phytozome.net). A complete list of the coding sequences (CDS), deduced amino acid sequences and genomic DNA sequences is available in Additional file 2.

*PSORT predictions: P (plasma membrane), V (vacuolar membrane), C (cytosol), Ch (chloroplast), N (nuclear), E.R. (endoplasmic reticulum), M (mitochondrion). The numbers indicate the number of nearest neighbors to the query which localize to each site.

To investigate the evolutionary relationships of Hsp90 proteins from different plants, we identified *Hsp90* genes from seven other plant species, including the moss *Physcomitrella patens*, the monocotyledonous angiosperms *Oryza sativa*, *Sorghum bicolor*, and *Brachypodium distachyon*, the dicotyledonous angiosperms *Arabidopsis thaliana*, *Vitis vinifera*, and *Medicago truncatula*. All angiosperm genomes, as well as the moss genome, contain *Hsp90* genes. The number of *Hsp90s* identified is seven in *A*. *thaliana*, ten in *P*. *trichocarpa*, five in *V*. *vinifera*, five in *M*. *truncatula*, eight in *O*. *sativa*, seven in *S*. *bicolor*, eight in *B*. *distachyon*, and ten in *P*. *patens*. Additional file 3 provides a complete list of all *Hsp90* genes identified in the present study.

### Phylogenetic analyses of the *Hsp90* gene family

**Figure 1:**
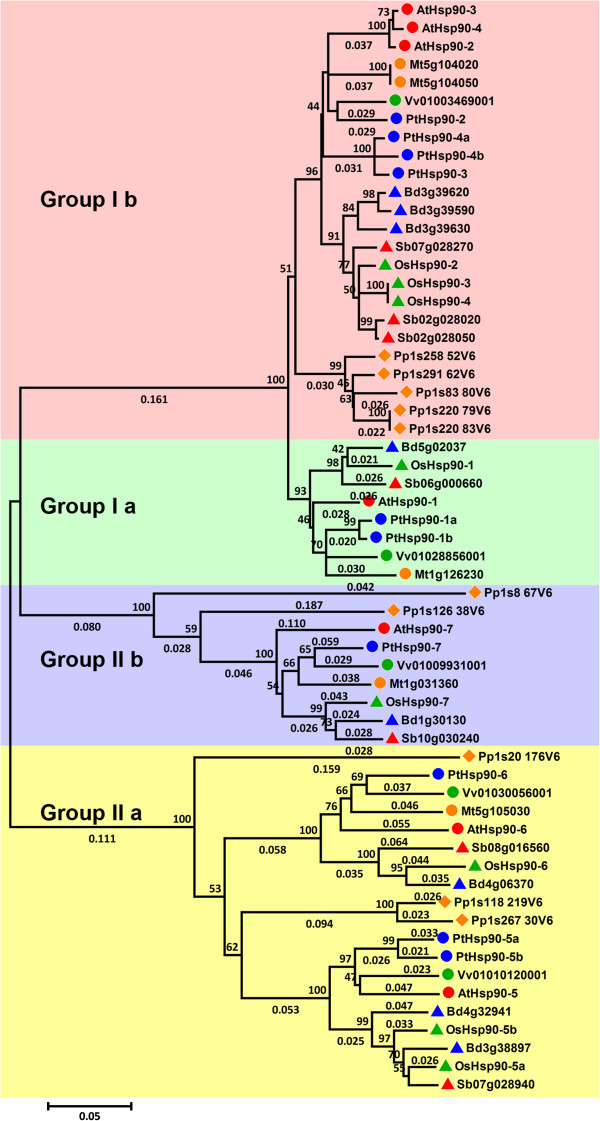
**Phylogenetic relationships of Hsp90 family members from eight plant species.** Multiple alignment of Hsp90 proteins from *A*. *thaliana* (At), *P*. *trichocarpa* (Pt), *O*. *sativa* (Os), *M*. *truncatula* (Mt), *S*. *bicolor*, (Sb), *B*. *distachyon* (Bd), *V*. *vinifera* (Vv), and *P*. *patens* (Pp) was performed using Clustal X2.1, and a phylogenetic tree was constructed using full-length protein sequences by the maximum likelihood method using PhyML. Bootstrap support values are shown on selected branches.

To examine the phylogenetic relationships among the *Hsp90* genes in *P*. *trichocarpa* and other plant species, we first generated a maximum likelihood phylogenetic tree by aligning full-length Hsp90 protein sequences from eight different plant species using PhyML. All of the sequences are classified into two major groups (group I and II), each of which is further divided into two subgroups (subgroup Ia, Ib, IIa and IIb) (Figure 1). The distribution of Hsp90 members in different species varies, and subgroups Ib and IIa are the largest two subgroups. There are two subgroup Ia members in *P*. *trichocarpa*, but none in moss and only one in the other species examined (Table 2). There are also more group Ib Hsp90 members in moss than these in the other species analyzed.

**Table 2:** Numbers of Hsp90s within each plant species

	**Ia**	**Ib**	**IIa**	**IIb**
*A. thaliana*	1	3	2	1
*O. sativa*	1	3	3	1
*P. trichocarpa*	2	4	3	1
*V. vinifera*	1	1	2	1
*M. truncatula*	1	2	1	1
*S. bicolor*	1	3	2	1
*B. distachyon*	1	3	3	1
*P. patens*	0	5	3	2

**Figure 2:**
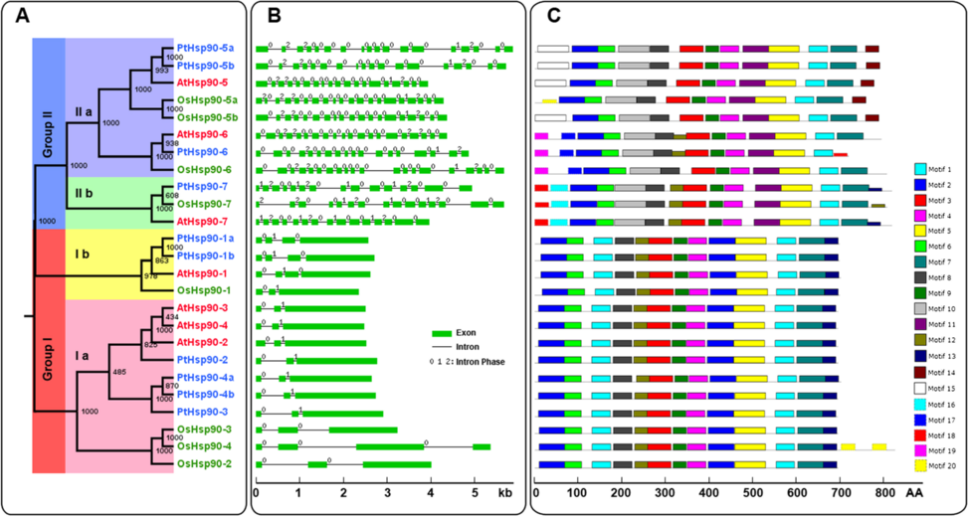
**Phylogenetic relationships, gene structures, and motif composition of *Hsp90 *genes in *A*. *thaliana *(At), *P*. *trichocarpa *(Pt), and *O*. *sativa *(Os). A**. A multiple alignment of full-length Hsp90 protein sequences from three species was executed using Clustal X2.1 and a phylogenetic tree was constructed using MEGA 4.0 by the neighbor-joining (NJ) method with 1000 bootstrap replicates. Bootstrap support is indicated at each node. The two major groups are marked with different background colors. **B**. Exon/intron structures of the *Hsp90* genes. Green boxes represent exons and black lines represent introns. The numbers indicate the splicing phases of the *Hsp90* genes: 0, phase 0; 1, phase 1; and 2, phase 2. **C**. Schematic representation of conserved motifs (obtained using MEME) in Hsp90 proteins. Different motifs are represented by boxes of different colors. Details of the individual motifs are shown in Additional file 5.

Next, we constructed a phylogenetic tree of the Hsp90 protein sequences from *Populus*, *Arabidopsis*, and rice using the neighbor-joining (NJ) method (Figure 2A). The tree topologies produced by two algorithms are largely comparable, with only minor differences at interior branches (Figure 2A). Distance and percentage of identity among *Populus*, *Arabidopsis*, and rice Hsp90 proteins are given in Additional file 4. Phylogenetic analysis shows that there is high similarity among the cytosolic members and less similarity among the organelle-type members. In addition, both trees show that the most recent duplicated pairs (Hsp90-1a/Hsp90-1b, Hsp90-4a/Hsp90-4b and Hsp90-5a/Hsp90-5b) exhibit high similarity, which indicates that they evolved slowly in sequence and structure, and may still keep their function.

It is more accurate to reflect an evolutionary relationship by using conserved domain sequences [18]. Therefore we also constructed the phylogenetic tree with the conserved Hsp90 motif sequences from *Populus*, *Arabidopsis*, and rice using the maximum likelihood method with 1000 bootstrap replicates (Additional file 6). The resulted phylogenetic tree is consistent with the one generated based on the full length protein sequences.

### Gene structure and conserved motifs of *Hsp90* genes in *Populus*, *Arabidopsis*, and rice

To further investigate the structural diversity of *Hsp90* genes in *Populus*, *Arabidopsis*, and rice, we first constructed a separate phylogenetic tree using the full-length Hsp90 protein sequences from these three species. The Hsp90 proteins are classified into two groups as described above (Figure 2A). Then we analyzed the exon/intron organization in the coding sequence of each *Hsp90* gene (Figure 2B). In general, the positions of some spliceosomal introns are conserved in orthologous genes. In many cases, conservation of exon/intron organization or gene structure in paralogous genes is high and sufficient to reveal the evolutionary relationship between introns [21]. In the present study, *Hsp90* gene family members within the same group shared similar gene structures in terms of intron number or exon length (Figure 2B). Hsp90 group I comprises the cytosolic Hsp90s whose members have two or three introns, while group II comprises organelle-type Hsp90s, which have 13–19 introns (Figure 2 and Table 1). The gene structure difference between group I and group II Hsp90 might associate with their functions in different biological processes in subcellular compartments. We also investigated intron phases with respect to codons. The intron phases are remarkably well conserved among group members, while the intron arrangements and intron phases are strikingly distinct between groups (Figure 2). This may lend support to the results of phylogenetic and genome duplication analyses. We further examined the exon/intron organization of paralogous pairs of *Hsp90* genes to explore traceable intron gain or loss within these genes. Three paralogous pairs in *Populus* (*PtHsp90-1a/1b*, *PtHsp90-4a/4b*, and *PtHsp90-5a/5b*) show conserved exon/intron structures in terms of intron number or gene length, while *OsHsp90-5a* shows a single intron gain event during the structural evolution of the *OsHsp90-5a/5b* paralogous pair. Interestingly, *OsHsp90-4* has an additional C-terminal exon compared to other members of the group I *Hsp90*.

Next, we predicted the major domains of these proteins in all three species using Pfam and CDD [18]. All of the proteins contain a HATPase_c superfamily domain and a Hsp90 family domain (Additional file 1). Although the tools we used are suitable for defining the presence or absence of recognizable domains, they are unable to recognize smaller individual motifs and more divergent patterns. Thus, the program MEME was used to further study the diversification of these proteins [22]. Twenty distinct motifs were identified (Figure 2C). Details of the 20 motifs are presented in Additional file 5. Most of the closely related members have common motif composition, suggesting possible functional similarity among these Hsp90 proteins (Figure 2C). Motif 2 and 6 (corresponding to the HATPase_c superfamily domain at the N-terminus) are found in all Hsp90 proteins from the species we examined. It has reported that both ATP binding and hydrolysis are required for Hsp90 function *in vivo*[23,24]. Noticeably, motif 20 (representing the LEA_6 subdomain) is only found in OsHsp90-4. This additional LEA_6 subdomain might explain the specific ability of OsHsp90-4 to acclimatize to various stresses.

### Chromosomal location and gene duplication of *Hsp90* genes in *Populus*, *Arabidopsis*, and rice

Chromosomal mapping of the gene loci shows that the 10 *PtHsp90* genes are distributed unevenly among nine chromosomes (Additional file 7). Two *PtHsp90* genes are localized on chromosome I, and one is localized on each of chromosome IV, V, VI, VIII, X, XIV, XVI, and XVII. Gene duplication events are thought to occur frequently in organismal evolution [25,26]. Previous studies report that the *Populus* genome has experienced at least two genome-wide duplication events (eurosid and salicoid), followed by a series of chromosomal reorganizations involving reciprocal tandem/terminal fusions and translocations [17]. To investigate the possible relationship between *Hsp90* genes and segmental chromosome duplication, we also compared the locations of *Hsp90* genes in duplicated chromosomal blocks that were previously identified in *Populus*, *Arabidopsis*, and rice [17,27,28]. Their distributions are shown in Additional file 7 (*Populus*), Additional file 8 (*Arabidopsis*), and Additional file 9 (rice). The results suggest that segmental duplication and transposition events are not the major factors that led to the expansion of the *Populus Hsp90* gene family. It may be that dynamic changes occurred following segmental duplication and led to the loss of many of the duplicated *Hsp90* genes.

A search for duplicated genes using the Plant Genome Duplication Database (PGDD; http://chibba.agtec.uga.edu/duplication/) revealed the existence of three gene pairs (*PtHsp90-1a/PtHsp90-1b*, *PtHsp90-3/PtHsp90-4b*, and *PtHsp90-5a/PtHsp90-5b*) in *P*. *trichocarpa* (Additional file 10A) and two pairs (*OsHsp90-2/OsHsp90-3* and *OsHsp90-5a/OsHsp90-5b*) in *O*. *sativa* (Additional file 10B). Interestingly, *PtHsp90-4a* was not assigned as a duplicated gene with *PtHsp90-3* and *PtHsp90-4b*, indicating that *PtHsp90-4a* had experienced intensive recombination events after the recent duplication with *PtHsp90-4b*, which led to the great divergence in its adjacent regions. Of the three *Hsp90* pairs in *Populus* that we examined, only one pair, *PtHsp90-5a/PtHsp90-5b*, remained in a conserved position in segmental duplicated blocks (Additional file 7), suggesting that only this paralogous pair survived during the evolutionary process after chromosome duplication event.

### Subcellular localization of *Populus* Hsp90 proteins

**Figure 3:**
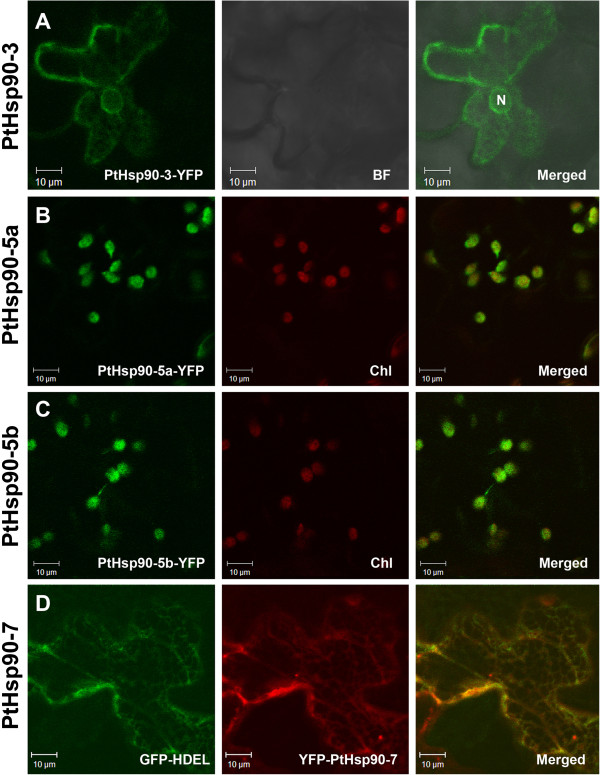
**Subcellular localization of PtHsp90 proteins. A**. Confocal image of an epidermal leaf cell expressing PtHsp90-3-YFP. **B**-**C**. Confocal images of epidermal leaf cells expressing PtHsp90-5a-YFP and PtHsp90-5b-YFP. The red channel shows autofluorescence of chlorophyll in photosynthetic tissues. **D**. Confocal images of epidermal leaf cells co-expressing YFP-PtHsp90-7 (red channel) and GFP-HDEL (green channel). Scale bar = 10 μm.

*In silico* analyses using the protein subcellular localization prediction software WoLF PSORT (http://wolfpsort.org) enabled us to predict the likely protein localization of each of the different candidate Hsp90s in *Populus*. PtHsp90-3 is predicted to be localized in the nucleus or in the cytosol with high reliability, while PtHsp90-5a and PtHsp90-5b are predicted to be localized in chloroplasts, PtHsp90-6 is predicted to be localized in mitochondria, and PtHsp90-7 is predicted to be localized in the ER. For the other PtHsp90 proteins, the cytosol is predicted to be their most likely location (Table 1). To confirm their predicted localizations, some of these proteins were transiently expressed in tobacco leaf epidermal cells as fusions with the N-terminus of YFP. Four Hsp90 proteins were successfully expressed as fluorescent protein fusions (PtHsp90-3-YFP, PtHsp90-5a-YFP, PtHsp90-5b-YFP, and YFP-PtHsp90-7). Based on sequence analysis, PtHsp90-1a, PtHsp90-1b, PtHsp90-2, PtHsp90-3, PtHsp90-4a, and PtHsp90-4b contain the C-terminal pentapeptide MEEVD (Additional file 2), which is characteristic of cytoplasmic Hsp90 proteins both in plants and in animals. In *Arabidopsis*, it was confirmed that two cytoplasmic Hsp90s (AtHsp90-1 and AtHsp90-3) are localized both in the nucleus and in the cytoplasm [29]. As shown in Figure 3A, the fluorescent signal of PtHsp90-3-YFP is also detected both in the nucleus and in the cytoplasm. This is consistent with the subcellular localizations of cytoplasmic *Arabidopsis* Hsp90 proteins [4,15]. Using the autofluorescence of chlorophyll as a marker, we found that the fluorescent signals of both PtHsp90-5a-YFP and PtHsp90-5b-YFP are well co-localized with red chlorophyll autofluorescence (Figure 3B and 3C). A transit peptide for the import into mitochondria was identified in the N-terminal region of PtHsp90-6, but the intercellular localization of PtHsp90-6 remains to be confirmed experimentally. The PtHsp90-7 protein sequence contains a C-terminal KDEL ER-retention motif (Additional file 2). When YFP-PtHsp90-7 is co-expressed with the well-characterized luminal ER marker GFP-HDEL [30], it is co-localized with GFP-HDEL (Figure 3D), which confirms its ER localization. These results suggest that the localization of Hsp90s in the same subgroup is relatively conserved among different species. The conserved organelle localization of Hsp90 implies that they might play roles in organelle-specific development or stress response. It has been suggested that mutation of the chloroplast-localized AtHsp90-5 causes altered response to red light, chlorate resistance and constitutively delayed chloroplast development in the *cr88* mutant [13,31,32]. In animals, a mitochondrial-localized Hsp90 appeared to have a critical role in cell cycle progression, cellular differentiation, and apoptosis [33,34]. In tobacco, mitochondrial-localized Hsp90 was involved in the *N* gene-dependent cell death by affecting downstream MAPK cascade function [35]. Mutation of the ER-localized AtHsp90-7 produced floral and shoot meristem phenotypes in the *shepherd* mutant that closely resemble that of the three *clavata* (*clv*) mutants in *Arabidopsis*[14,36,37]. The conserved subcellular localization of Hsp90s might provide clues for their specific cellular functions.

### Differential expression patterns of *Hsp90* genes in *Populus*

**Figure 4:**
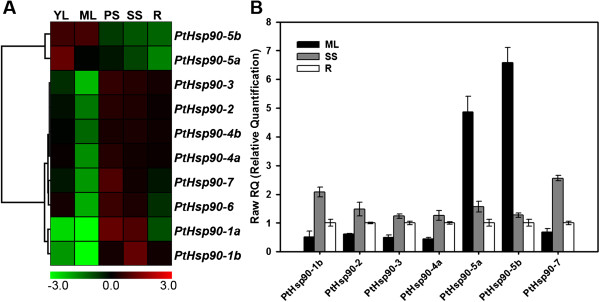
**Expression analysis of *PtHsp90 *genes in different tissues. A**. Heat map showing hierarchical clustering of *PtHsp90* genes in vegetative tissues (YL, young leaves; ML, mature leaves; PS, primary stem; SS, secondary stem; R, roots). The data was obtained from our unpublished RNA-seq data. The expression level of genes was determined based on the value of RPKM (reads per kilobase of exon region in a gene per million mapped reads). Details of the RPKM are shown in Additional file 11. Color scale represents log2 expression values. **B**. Expression of seven selected *PtHsp90s* was quantified by quantitative reverse-transcription polymerase chain reaction (qRT-PCR) in vegetative tissues (ML, mature leaves; SS, secondary stem; R, roots). The average expression of each gene was calculated relatively to the first biological replicate of roots ± standard error (SE) (n≥3).

The expression patterns of genes can provide useful clues for the functions of these genes. To verify the expression profiles of *Populus Hsp90* genes, the RNA-seq data of different *Populus* vegetative tissues (unpublished data) were used to analyze the expression of *PtHsp90* genes. *PtHsp90-5a* and *PtHsp90-5b* are mainly expressed in the young leaves (YL) and mature leaves (ML) (Figure 4A), which is consistent with their localization in chloroplasts (Figures 3B and 4C). The expression of *PtHsp90-5a* in the young leaves is stronger than that in the mature leaves, suggesting *PtHsp90-5a* may play roles in young leaf development. The other *PtHsp90* genes are mainly expressed in stems including primary stem (PS) or secondary stem (SS). *PtHsp90-1b* is highly expressed in secondary stem, while *PtHsp90-1a*, *PtHsp90-6* and *PtHsp90-7* are mainly expressed in primary stem. These results imply that these *PtHsp90s* might be involved in different stages of stem development. The transcription levels of *PtHsp90-2*, *PtHsp90-3*, *PtHsp90-4a* and *PtHsp90-4b* are higher than these of the other *PtHsp90* members. *PtHsp90-4a* and *PtHsp90-4b* are ubiquitously highly expressed in almost all detected tissues (Additional file 11). In order to verify the expression profiles of *PtHsp90* genes obtained by RNA-seq, qRT-PCR analysis of seven selected *PtHsp90* genes was performed on three different tissues (Figure 4B). The average expression of each gene was calculated relatively to the value of the first replication of roots ± standard error (SE) (n≥3). The gene expression pattern detected by qRT-PCR is generally consistent with the RNA-seq results. The different expression patterns of *PtHsp90s* in different tissues imply that PtHsp90 members may be involved in different biological processes.

### Differential stress responses of *Hsp90* genes in *Populus*

**Figure 5:**
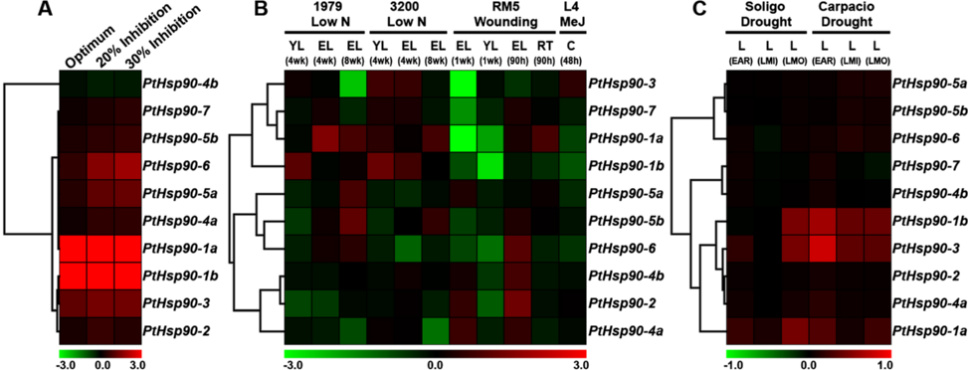
**Expression profiles of *PtHsp90 *genes. A**. Heat map showing 10 *PtHsp90* genes under heat stress. Expression is indicated as the fold-change in photosynthetic optimum (31.75°C, temperature producing the maximum net CO_2_ assimilation rate), 20% inhibition of optimum (38.4°C) and 30% inhibition of optimum (40.5°C) relative to baseline (22°C, the growth temperature), and was visualized using heat maps (see Methods). Microarray data were obtained from GEO (GSE26199). **B**. Heat map showing 10 *PtHsp90* genes across various tissues and genotypes (GEO: GSE16786). The genotypes analyzed were *P*. *fremontii × P*. *angustifolia* clones 1979, 3200, and RM5; *P*. *tremuloides* clones 271 and L4; and *P*. *deltoids* clones Soligo and Carpaccio. The tissues analyzed were young leaves (YL), expanding leaves (EL), mature leaves (L), root tips (RT), and suspension cell cultures (C). Stress treatments were nitrogen limitation (low N), methyl jasmonate elicitation (MeJ), and wounding, with sampling either 1 week or 90 h after wounding. **C**. Heat map showing 10 *PtHsp90* genes under conditions of short- and long-term water deficit (GEO: GSE17230). EAR, early response to water deficit (by 36 h), LMI, long-term (10-day) response to mild stress with a soil relative extractable water (REW) level of 20–35%, LMO, long-term (10-day) response to moderate stress with a soil REW level of 10–20%.

In order to reveal the responses of *Populus Hsp90* genes to abiotic stresses, we analyzed the expression profiles of *PtHsp90s* under abiotic stresses such as heat, low nitrogen levels, mechanical wounding, drought, and methyl jasmonate (MeJ) treatment. Affymetrix microarray data (series accession numbers GSE26199, GSE16786 and GSE17230 in the Gene Expression Omnibus [GEO]) [38] were used to analyze the global expression profiles of *Populus Hsp90* genes. Previous study divided the physiological condition into four states according to *Populus* photosynthetic activity from 22°C to 42°C: baseline (22°C, the growth temperature), optimum (31.75°C, temperature producing the maximum net CO_2_ assimilation rate), 20% inhibition of optimum (38.4°C) and 30% inhibition of optimum (40.5°C) [39]. Most *PtHsp90* genes are upregulated under heat stress. The expression of *PtHsp90-1a* and *PtHsp90-1b* is highly induced immediately when temperature increases to optimum. *PtHsp90-5a* and *PtHsp90-6* are highly induced when the photosynthesis is inhibited by 30% under heat stress (Figure 5A). In *PtHsp90* group I, *PtHsp90-1a*, *PtHsp90-1b*, and *PtHsp90-3* in both the Soligo and Carpacio genotypes are upregulated under almost all drought stresses tested, including the early response (EAR) to drought at 36 h, and the long-term (10 days) responses to mild stress (LMI) and moderate stress (LMO) (Figure 5C). Nitrogen deficiency stress causes different responses among *Hsp90* genes. For instance, *PtHsp90-1a* and *PtHsp90-1b* are upregulated in 4-week-old young leaves (YL) and 8-week-old expanded leaves (EL) of genotype 1979 and genotype 3200; *PtHsp90-5a* and *PtHsp90-5b* are upregulated in 8-week-old expanded leaves (EL) of the same two genotypes. However, *PtHsp90-3*, *PtHsp90-4a*, and *PtHsp90-6* are downregulated in 8-week-old expanded leaves in genotype 1979 and/or genotype 3200 (Figure 5B). In response to mechanical wounding stress, six genes (*PtHsp90-1a*, *PtHsp90-1b*, *PtHsp90-3*, *PtHsp90-5b*, *PtHsp90-6*, and *PtHsp90-7*) are significantly downregulated in young leaves and/or expanding leaves 1 week after wounding. In response to MeJ feeding in cell culture, only *PtHsp90-1a* and *PtHsp90-1b* are slightly downregulated (Figure 5B).

**Figure 6:**
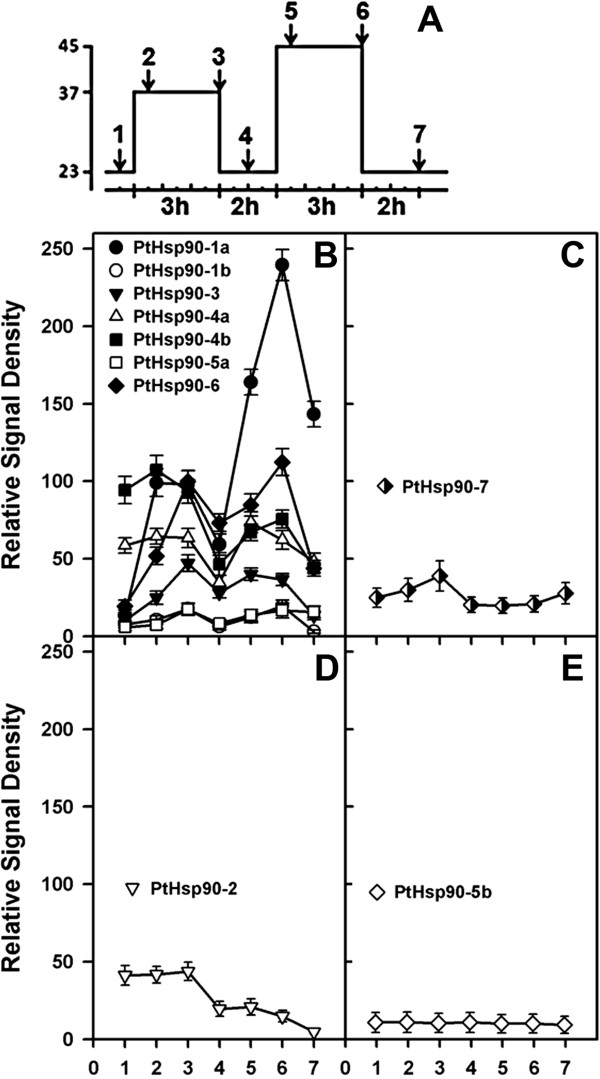
**Expression analysis of *PtHsp90 *genes under heat stress. A**. Conditions of heat stress. Seedlings were heated to 37°C for 3 h (pretreatment), returned to 23°C for 2 h, heated to 45°C for 3 h (treatment), and then allowed to recover for 2 h. **1**, control; **2**, 30 min after pretreatment at 37°C; **3**, 2 h after pretreatment at 37°C; **4**, 1 h after recovery at 23°C; **5**, 30 min after treatment at 45°C; **6**, 2 h after treatment at 45°C; **7**, 2 h after recovery at 23°C. **B-E.** The corresponding relative densities of the RT-PCR signals generated from data in Additional file 12B, showing four different types of heat stress responses. Type I is induced by both pretreatment at 37°C and treatment at 45°C **(B)**; Type II is induced by pretreatment at 37°C but is not affected by treatment at 45°C **(C)**; Type III is not affected by pretreatment at 37°C but is negatively regulated by treatment at 45°C **(D)**; and type IV is not affected by either pretreatment at 37°C or treatment at 45°C **(E)**.

The responses of *PtHsp90* genes to heat stress were analyzed experimentally. Heat-stress treatment comprising pretreatment for 3 h at 37°C and subsequent treatment at 45°C for 3 h, with a 2-h recovery interval, was performed. Most genes are induced by heat stress (Figure 6, Additional file 12). We classified the *PtHsp90s* into four classes according to their expression profiles under heat stress. Class I genes are induced immediately by both 37°C pretreatment and 45°C treatment (*PtHsp90s* in this class are positively regulated under both 37°C pretreatment and subsequent treatment at 45°C) (Figure 6B). Notably, *PtHsp90-1a* is induced 30 min after 37°C pretreatment and significantly induced 3 h after 45°C treatment in leaves. Class II genes are induced by 37°C pretreatment but not affected by 45°C treatment. *PtHsp90-7* belongs to this class and its expression is induced by 37°C pretreatment. However, the expression of *PtHsp90-7* is not affected by 45°C treatment following 2 h of recovery from 37°C pretreatment (Figure 6C). Class III genes are not affected by 37°C pretreatment but are negatively regulated by 45°C treatment. *PtHsp90-2* is not induced by 37°C pretreatment, and its mRNA abundance is reduced after recovery from 37°C pretreatment and subsequent 45°C treatment (Figure 6D). Class IV genes are not affected by either 37°C pretreatment or 45°C treatment significantly. The expression of *PtHsp90-5b* is still maintained in a low level in 37°C pretreatment and 45°C treatment (Figure 6E).

To verify the expression profiles of *PtHsp90* genes in response to heat stress, qRT-PCR analysis was performed for four selected *PtHsp90* genes under heat stress (Additional file 12C-F). Notably, *PtHsp90-3*, *PtHsp90-4a* and *PtHsp90-5a* are induced 3 h after 37°C pretreatment and significantly induced 3 h after 45°C treatment in leaves (Additional file 12C-E). The expression of *PtHsp90-5b* is also induced by heat stress, but the induction is not that dramatic compared with that of the other *PtHsp90* genes in both 37°C pretreatment (2-fold) and 45°C treatment (3-fold) (Additional file 12F). In addition, we found that the paralogous pair *PtHsp90-5a*/*PtHsp90-5b* shared the same expression profile in different tissues but were different under wounding and heat stresses (Figures 5 and 6). We then analyzed the promotors (2000 bp upstream of the start codon) of *PtHsp90-5a* and *PtHsp90-5b* using PlantCARE [40]. The sequence of the promotors share a low sequence identity (43.3%) and two heat shock elements (HSE) exist in the promotor of *PtHsp90-5a* while none in *PtHsp-5b* (data not shown), which may contribute to the different expression pattern of the two genes. These results suggest different response mechanisms of *PtHsp90* members may exist under heat stress, and provide significant insights into their functions.

## Conclusions

We performed a comprehensive analysis of the *Populus Hsp90* gene family covering phylogeny, chromosomal location, gene structure, subcellular localization, expression profiling, and heat stress responses. A total of 10 full-length *Hsp90* genes were identified in the *Populus* genome, all of which are clustered into two distinct groups. Exon/intron structure and motif compositions are found to be relatively conserved in each subgroup. The *Populus* genome contains three paralogous *Hsp90* gene pairs, but only *PtHsp90-5a/PtHsp90-5b* is located in conserved positions in duplicated blocks, suggesting that it may be derived from a segmental duplication event during evolution. Furthermore, subcellular localization analysis revealed that PtHsp90 members are localized in different organelles. In addition, comparative expression profile analysis of *Populus Hsp90s* revealed that Hsp90s may play various conserved roles in different biological processes in plants. Although the functions of *PtHsp90s* remain largely unknown and many experiments are needed to determine their precise functions, our phylogenetic and expression analyses of the *Populus Hsp90* gene family establishes a solid foundation for future comprehensive functional analyses of *PtHsp90s*.

## Methods

### Database searching and sequence retrieval

To identify potential members of the *Populus Hsp90* gene family, we performed multiple database searches. Published *Arabidopsis Hsp90* gene sequences [4] were retrieved and used as queries in BLAST searches against the Poplar Genome Database (http://www.phytozome.net/poplar.php, release 3.0). BLAST searches were also performed against the poplar genomes at the National Center for Biotechnology Information (NCBI, http://www.ncbi.nlm.nih.gov) and Phytozome (http://www.phytozome.net). Rice *Hsp90* gene sequences were downloaded from the Rice Genome Annotation Project Database (http://rice.plantbiology.msu.edu/, release 7). Sequences of *M*. *truncatula*, S. *bicolor*, *B*. *distachyon*, *V*. *vinifera*, and *P*. *patens* were downloaded from Phytozome (http://www.phytozome.net). Local BLAST searches were performed using *Arabidopsis* Hsp90 protein sequences as queries to identify Hsp90 sequences in these plant species. All of the sequences were manually analyzed to confirm the presence of HATPase and Hsp90 domains using InterProScan (http://www.ebi.ac.uk/Tools/pfa/iprscan/).

WoLF PSORT (http://wolfpsort.org) was used to predict protein subcellular localization. The pI and molecular weight were estimated using the Compute pI/Mw tool from ExPASy (http://web.expasy.org/compute_pi).

Functional motifs or domains of PtHsp90 sequences were analyzed using the PROSITE (http://prosite.expasy.org/) and Conserved Domain databases (http://www.ncbi.nlm.nih.gov/Structure).

### Phylogenetic analyses

Multiple sequence alignment of the full-length protein sequences was performed using ClustalX2 (version 2.1) [41]. A maximum likelihood (ML) phylogenetic tree was constructed using PhyML (v3.0) with the JTT amino acid substitution model, 1000 bootstrap replicates, estimated proportions of invariable sites, four rate categories, estimated gamma distribution parameters, and an optimized starting BIONJ tree [42,43].

### Chromosomal location and gene structure of the *Hsp90* genes

The chromosomal locations of the *Hsp90* genes were determined using the *Populus* genome browser (http://www.phytozome.net/poplar). Information on intron/exon structure was collected from the genome annotations of *P*. *trichocarpa* from NCBI and Phytozome.

### Gene structure analysis

The exon and intron structures of individual *Hsp90* genes were illustrated using Gene Structure Display Server (GSDS, http://gsds.cbi.pku.edu.cn/) [44] by aligning the cDNA sequences with the corresponding genomic DNA sequences from Phytozome.

### Conserved motif analysis

Functional motifs or domains of PtHsp90 protein sequences were analyzed using PROSITE and the Conserved Domain database. MEME (http://meme.sdsc.edu) [22] was used to identify motifs in candidate sequences. MEME was run locally with the following parameters: number of repetitions = any, maximum number of motifs = 20, and optimum motif width = 30 to 70 residues.

### Transient expression and imaging

Transient expression in *Nicotiana benthamiana* lower leaf epidermal cells was performed as described by Zheng et al. [45] with slight modifications. Plants were cultivated under short-day conditions (8 h light/16 h dark). When the agrobacterium culture reached the stationary growth phase at 28°C with agitation, cells were pelleted and resuspended in infiltration buffer (100 μM acetosyringone in 10 mM MgCl_2_).

### Publicly available microarray data analyses

For abiotic and hormonal treatments, Affymetrix microarray data available in the NCBI GEO database under the series accession numbers GSE26199 (heat stress), GSE17230 (drought stress) and GSE16786 were analyzed [39,46,47]. GSE16786 is composed of the following five subsets: GSE14893 (nitrogen limitation, genotype 1979), GSE14515 (nitrogen limitation, genotype 3200), GSE16783 (1 week after leaf wounding), GSE16785 (90 h after leaf wounding), and GSE16773 (methyl jasmonate-elicited suspension cell cultures). The Affymetrix CEL files representing different abiotic and hormonal treatments were downloaded from the GEO database and preprocessed using GeneSpring GX (V11.5) software (Agilent Technologies). The data were normalized using the GCRMA algorithm and then log transformed. The averages were calculated. After normalization and log transformation of data for all of the *Populus* genes presented on the chip, the log signal intensity values for *Populus* probe IDs corresponding to the *Hsp90* gene models (v1.1) were extracted as a subset for further analyses. Expression was shown as fold change in experimental treatment samples relative to control samples. Tab-delimited files for the average log signal intensity values were imported into Genesis (v1.75) to generate the heat maps [48].

Probe sets corresponding to *PtHsp90* genes were identified using the online Probe Match tool POParray (http://aspendb.uga.edu/poparray). For probe sets matching several *Populus Hsp90* gene models, only these exhibiting consistently high hybridization signals across multiple samples were considered.

### Plant material and growth conditions

Plant materials were collected from clonally propagated 1-year-old hybrid poplar (*P*. *alba* × *P*. *glandulosa*) clones (84K) grown in a growth chamber under long-day conditions (16 h light/8 h dark) at 23–25°C. Poplar saplings were subjected to heat treatment. Briefly, chamber was heated to 37°C for 3 h (pretreatment), returned to 23°C for 2 h, heated to 45°C for 3 h (treatment), and then allowed to recover for 2 h. Two biological replicates were performed. Leaves from three different plants were harvested at seven selected time points during heat stress treatment, frozen immediately in liquid nitrogen, and stored at −80°C for further analysis.

### RNA isolation and semi-quantitative RT-PCR

Total RNA was extracted using the RNeasy Plant Mini Kit (Qiagen) with on-column treatment with RNase-free DNase I (Qiagen) to remove any contamination of genomic DNA according to the manufacturer’s instructions. RNA integrity was verified by 2% agar gel electrophoresis. First-strand cDNA synthesis was carried out with approximately 1 μg RNA using the SuperScript III reverse transcription kit (Invitrogen) and random primers according to the manufacturer’s procedure. Primers with melting temperatures of 58–60°C, lengths of 20–27 bp, and amplicon lengths of 160–260 bp were designed using Primer3 software (http://frodo.wi.mit.edu/primer3/input.htm). All primer sequences are listed in Additional file 13.

Real-time PCR was conducted on 7500 Real Time PCR System (Applied Biosystems, CA, USA) using SYBR Premix Ex Taq™ Kit (TaKaRa, Tokyo, Japan). Reactions were prepared in a total volume of 20 μl containing: 10 μl of 2×SYBR Premix, 2 μl of cDNA template, 0.4 μl of each specific primer to a final concentration of 200 nM. The reactions were performed as the following conditions: initial denaturation step of 95°C for 30 s followed by two-step thermal cycling profile of denaturation at 95°C for 10 s, and combined primer annealing/extension at 60°C for 34 s for 40 cycles. Negative PCR control without templates was performed for each primer pair. To verify the specificity of each primer pair, a melting curve analysis was performed ranging from 60°C to 95°C with temperature increasing steps of 0.06°C/s (5 acquisitions per °C) at the end of each run. The final threshold cycle (Ct) values were the mean of eight values including two biological replicates for each treatment and four technical replicates. The *PtActin* gene was used as an internal control.

## Competing interest

The authors declare that they have no competing interests.

## Authors’ contributions

JZ carried out all the analysis and interpreted the results. JL, LZ and BL helped in *Populus* materials collection and total RNA extraction. ML and JC conceived the project, supervised the analysis and critically revised the manuscript. All authors read and approved the final manuscript.

## Supplementary Material

Additional file 1 **Conserved domains of Hsp90 proteins in *Arabidopsis, Populus*, and rice.** The major domains were identified using Pfam (http://pfam.sanger.ac.uk/). A multiple alignment of Hsp90 proteins from *Arabidopsis* (At), *Populus* (Pt), and rice (Os) was performed using Clustal X2.1, and a phylogenetic tree was constructed using MEGA 4.0 by the neighbor-joining (NJ) method with 1000 bootstrap replicates. Click here for file

Additional file 2 **List of all *Hsp90 *gene sequences identified in *Populus *and rice.** The list comprises seven *Arabidopsis* Hsp90 sequences and Hsp90 sequences identified from *Populus* and rice in this study. Amino acid sequences were deduced from their corresponding coding sequences, and genomic DNA sequences were obtained from Phytozome (http://www.phytozome.net/poplar, release 2.1). Click here for file

Additional file 3 List of Hsp90 protein sequences identified from eight plant species examined in this study. Click here for file

Additional file 4 **Distance and percentage of identity among *Arabidopsis, Populus***,** and rice Hsp90 proteins.** Amino acid identity among *Populus*, *Arabidopsis*, and rice Hsp90 proteins was analyzed in a pairwise fashion. Click here for file

Additional file 5 **Sequence logos for the conserved motifs of Hsp90 proteins in *Arabidopsis, Populus*, and rice.** Conserved motifs and sequence logos were generated using the MEME search tool. Numbers on the horizontal axis represent sequence positions in the motifs and the vertical axis represents the information content in bits. Click here for file

Additional file 6 **Phylogenetic relationships of Hsp90 conserved motif sequences in *Arabidopsis, Populus***,** and rice.** A multiple alignment of Hsp90 proteins from *A*. *thaliana* (At), *P*. *trichocarpa* (Pt) and *O*. *sativa* (Os) was performed using Clustal X2.1, and a phylogenetic tree was constructed using conserved Hsp90 motif sequences by the maximum likelihood method with 1000 bootstrap replicates. Click here for file

Additional file 7 **Chromosomal locations of *PtHsp90 *genes.** The schematic diagram shows the 10 *Hsp90* genes mapped to nine chromosomes. Homologous blocks derived from segmental duplication are indicated using the same colors. The diagram of genome-wide chromosome organization resulting from genome duplication events in *Populus* is adapted from Tuskan et al. [17]. Click here for file

Additional file 8 **Chromosomal locations of *Arabidopsis Hsp90* genes.** The lines join the segmental duplicated homologous blocks. Click here for file

Additional file 9 **Chromosomal locations of rice *Hsp90* genes.** The lines join the segmental duplicated homologous blocks that are indicated using the same colors. Click here for file

Additional file 10 **Gene duplication relationships in the *Hsp90 *gene family in *Populus trichocarpa *and *Oryza sativa*.** Paralogous gene pairs generated by gene duplication within the *Hsp90* family of *P*. *trichocarpa* (A) and *O*. *sativa* (B) were analyzed using the Plant Genome Duplication Database (http://chibba.agtec.uga.edu/duplication/). Each query gene displays only ±500 kb regions. Gene lines connect gene pairs. Blue lines represent the other anchor gene pairs in the region, and the red line represents the query locus. Click here for file

Additional file 11 **RPKM of *PtHsp90 *genes in vegetative tissues obtained from RNA-seq data.** Click here for file

Additional file 12 **Expression analysis of selected *PtHsp90 *genes under heat stress.** A. Conditions of heat stress. Seedlings were heated to 37°C for 3 h (pretreatment), returned to 23°C for 2 h, heated to 45°C for 3 h (treatment), and then allowed to recover for 2 h. 1, control; 2, 30 min after pretreatment at 37°C; 3, 2 h after pretreatment at 37°C; 4, 1 h after recovery at 23°C; 5, 30 min after treatment at 45°C; 6, 2 h after treatment at 45°C; 7, 2 h after recovery at 23°C. B. Analysis of expression profiles of *PtHsp90s* in response to heat stress in *Populus* leaves by semi-quantitative RT-PCR. The constitutively expressed *PtActin* was used as an internal control. Three independent experiments were performed under identical conditions. C-F. The relative mRNA abundance of four selected *PtHsp90* genes was normalized with respect to reference gene *PtActin* under heat stress using qRT-PCR. Three biological replicates each with four technique replicates were performed and bars represent standard deviations (SD) of the replicates. Click here for file

Additional file 13 **Primers used in RT-PCR analysis of the 10 *PtHsp90 *genes.** Click here for file
