# Supplementary figures and images for: Genome-wide analysis of the Populus Hsp90 gene family reveals differential expression patterns, localization, and heat stress responses
Source: BMC Genomics. 2013 Aug 5;14:532. doi: 10.1186/1471-2164-14-532 (PMC3750472; doi:10.1186/1471-2164-14-532)

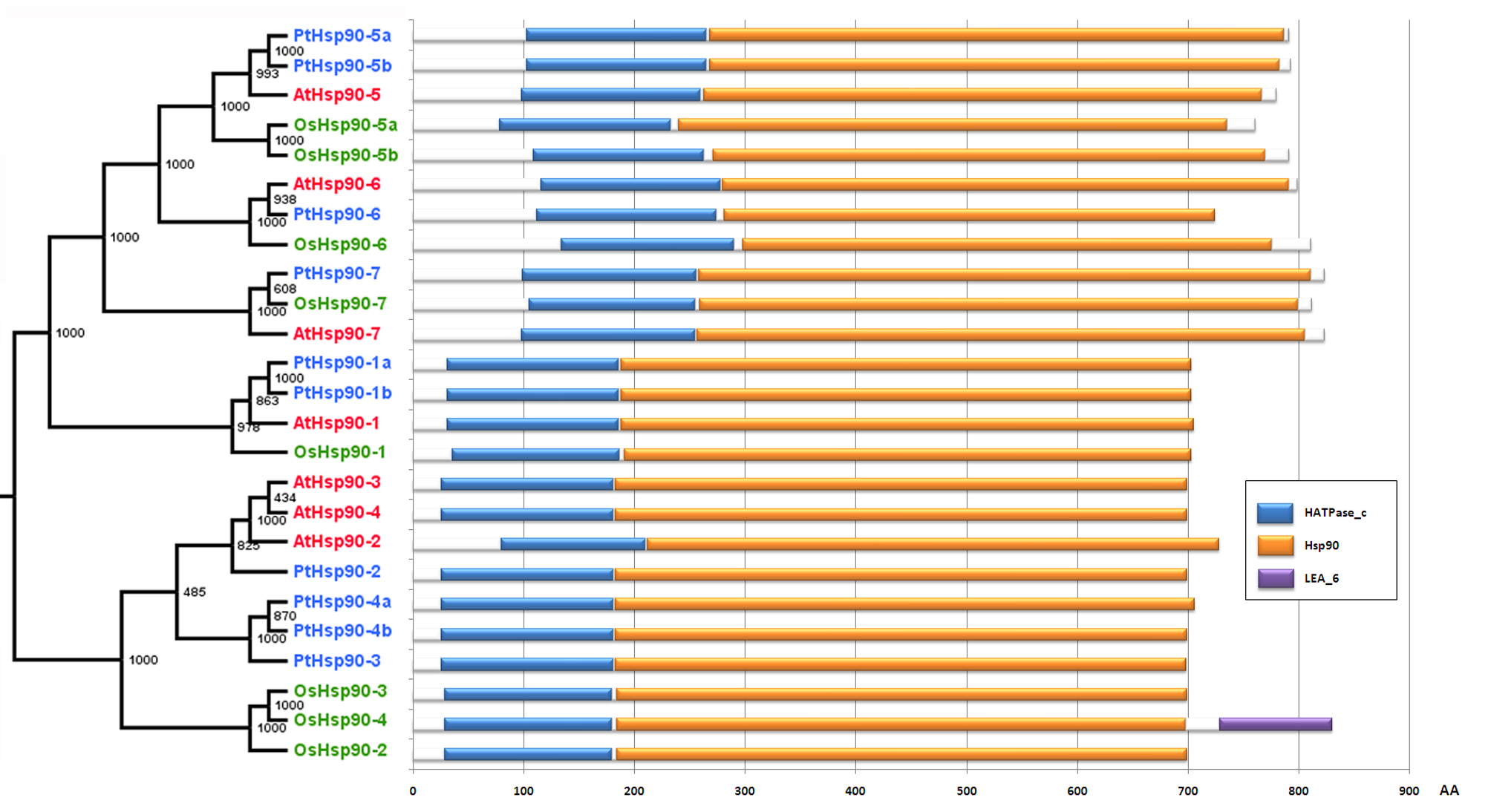

Supplement: Additional file 1 — Conserved domains of Hsp90 proteins in Arabidopsis, Populus, and rice. The major domains were identified using Pfam (http://pfam.sanger.ac.uk/). A multiple alignment of Hsp90 proteins from Arabidopsis (At), Populus (Pt), and rice (Os) was performed using Clustal X2.1, and a phylogenetic tree was constructed using MEGA 4.0 by the neighbor-joining (NJ) method with 1000 bootstrap replicates. [file 1471-2164-14-532-S1.jpg]

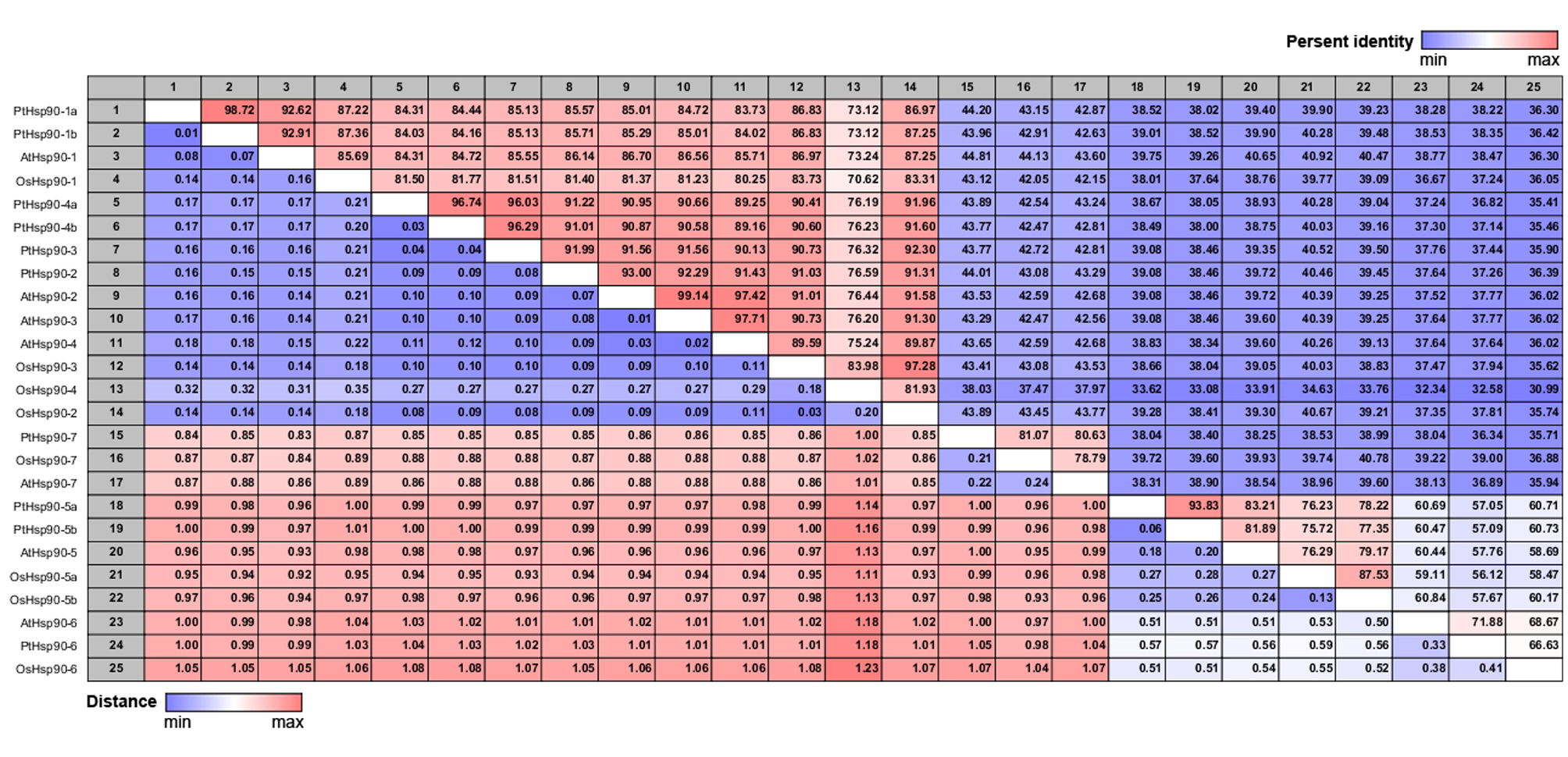

Supplement: Additional file 4 — Distance and percentage of identity among Arabidopsis, Populus, and rice Hsp90 proteins. Amino acid identity among Populus, Arabidopsis, and rice Hsp90 proteins was analyzed in a pairwise fashion. [file 1471-2164-14-532-S4.jpg]

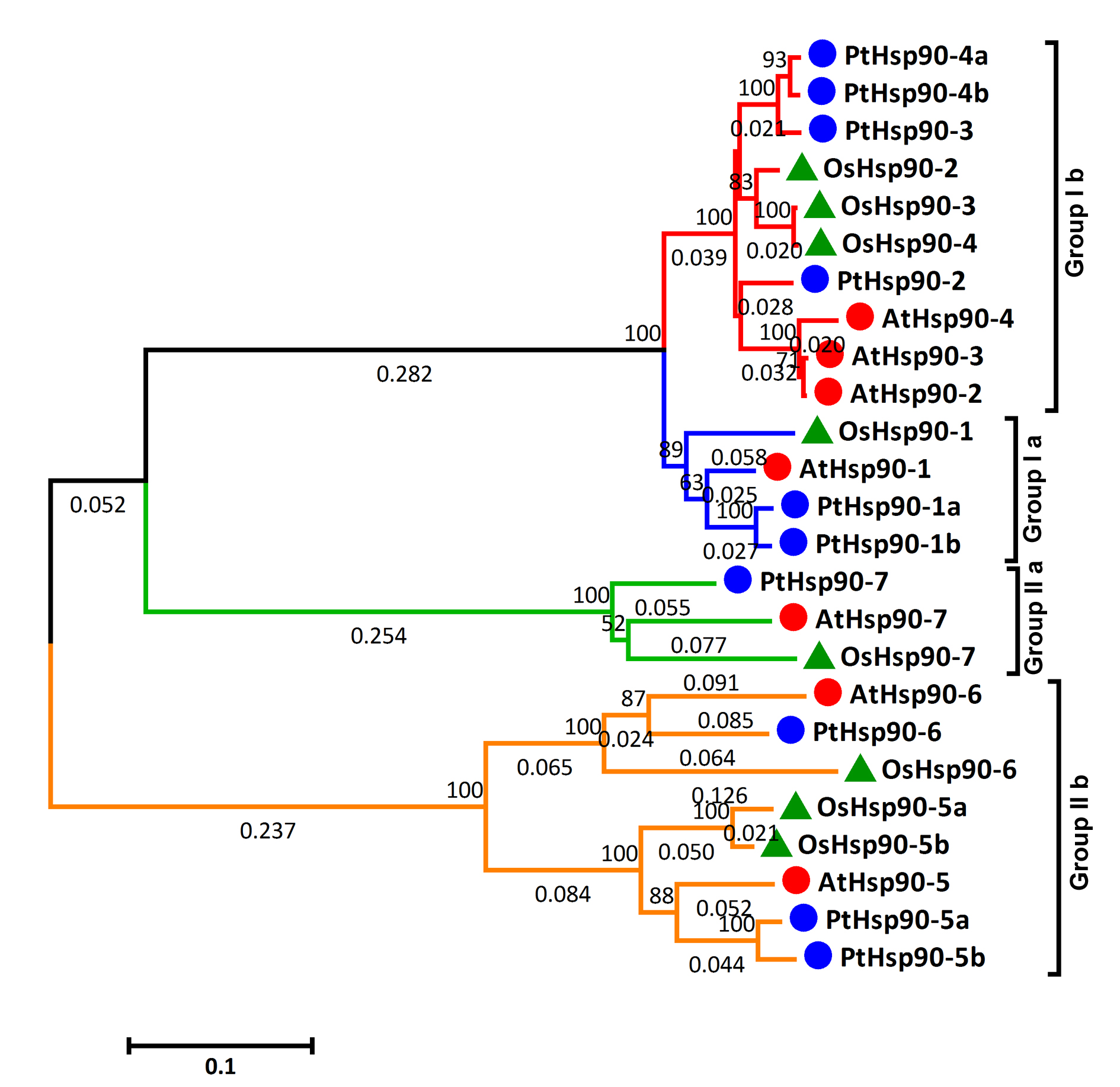

Supplement: Additional file 6 — Phylogenetic relationships of Hsp90 conserved motif sequences in Arabidopsis, Populus, and rice. A multiple alignment of Hsp90 proteins from A. thaliana (At), P. trichocarpa (Pt) and O. sativa (Os) was performed using Clustal X2.1, and a phylogenetic tree was constructed using conserved Hsp90 motif sequences by the maximum likelihood method with 1000 bootstrap replicates. [file 1471-2164-14-532-S6.jpg]

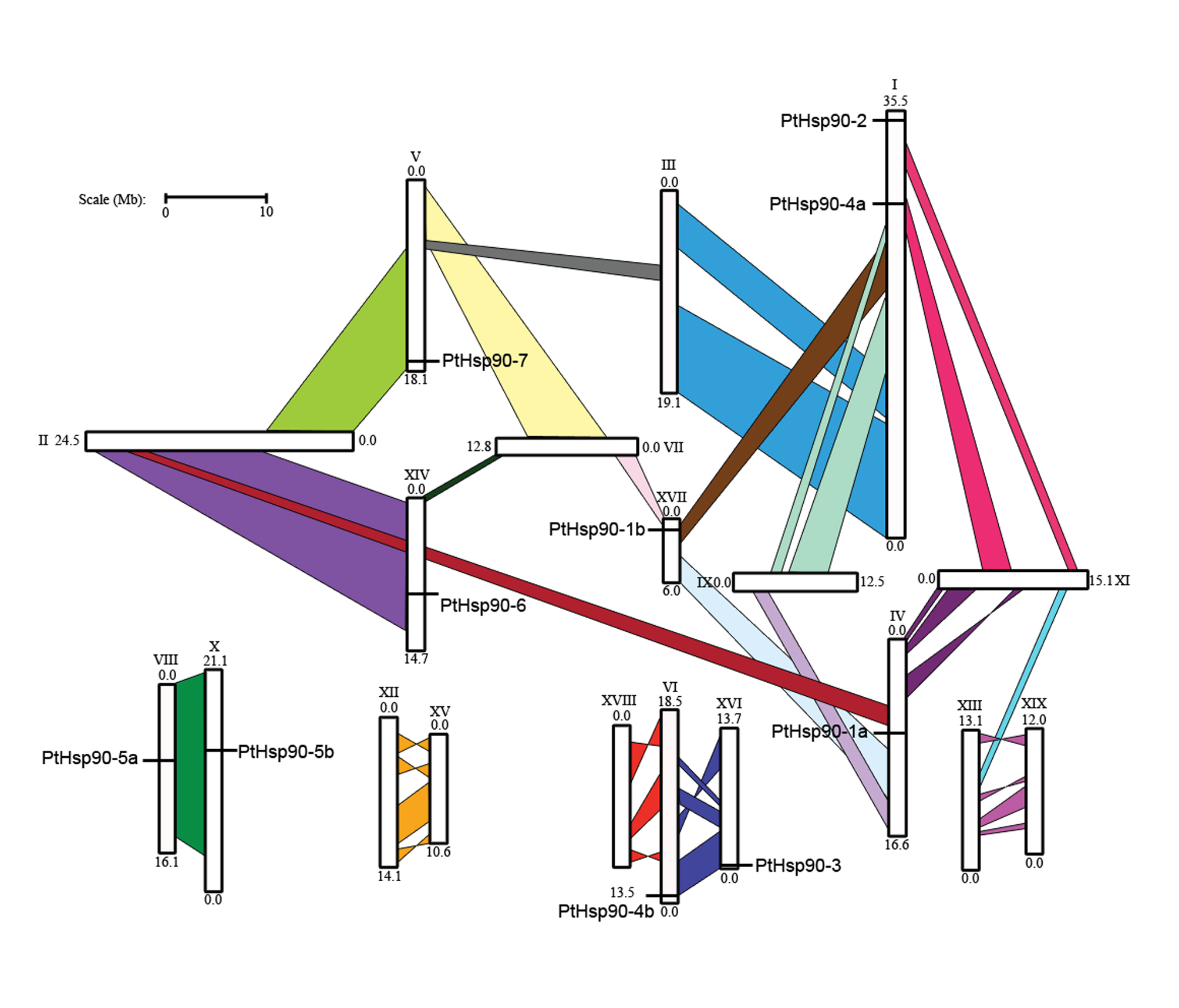

Supplement: Additional file 7 — Chromosomal locations of PtHsp90 genes. The schematic diagram shows the 10 Hsp90 genes mapped to nine chromosomes. Homologous blocks derived from segmental duplication are indicated using the same colors. The diagram of genome-wide chromosome organization resulting from genome duplication events in Populus is adapted from Tuskan et al. [17]. [file 1471-2164-14-532-S7.jpg]

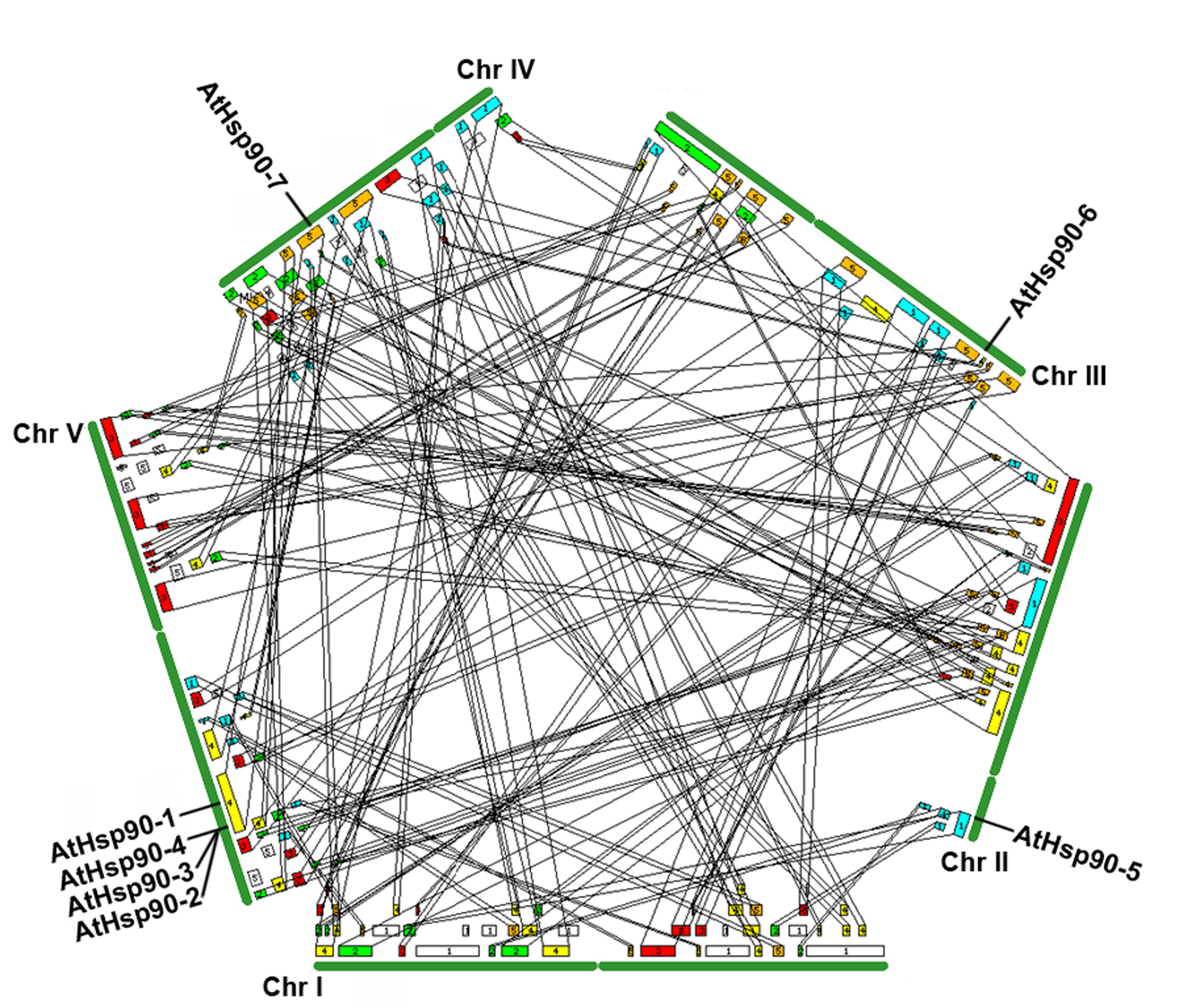

Supplement: Additional file 8 — Chromosomal locations of Arabidopsis Hsp90 genes. The lines join the segmental duplicated homologous blocks. [file 1471-2164-14-532-S8.jpg]

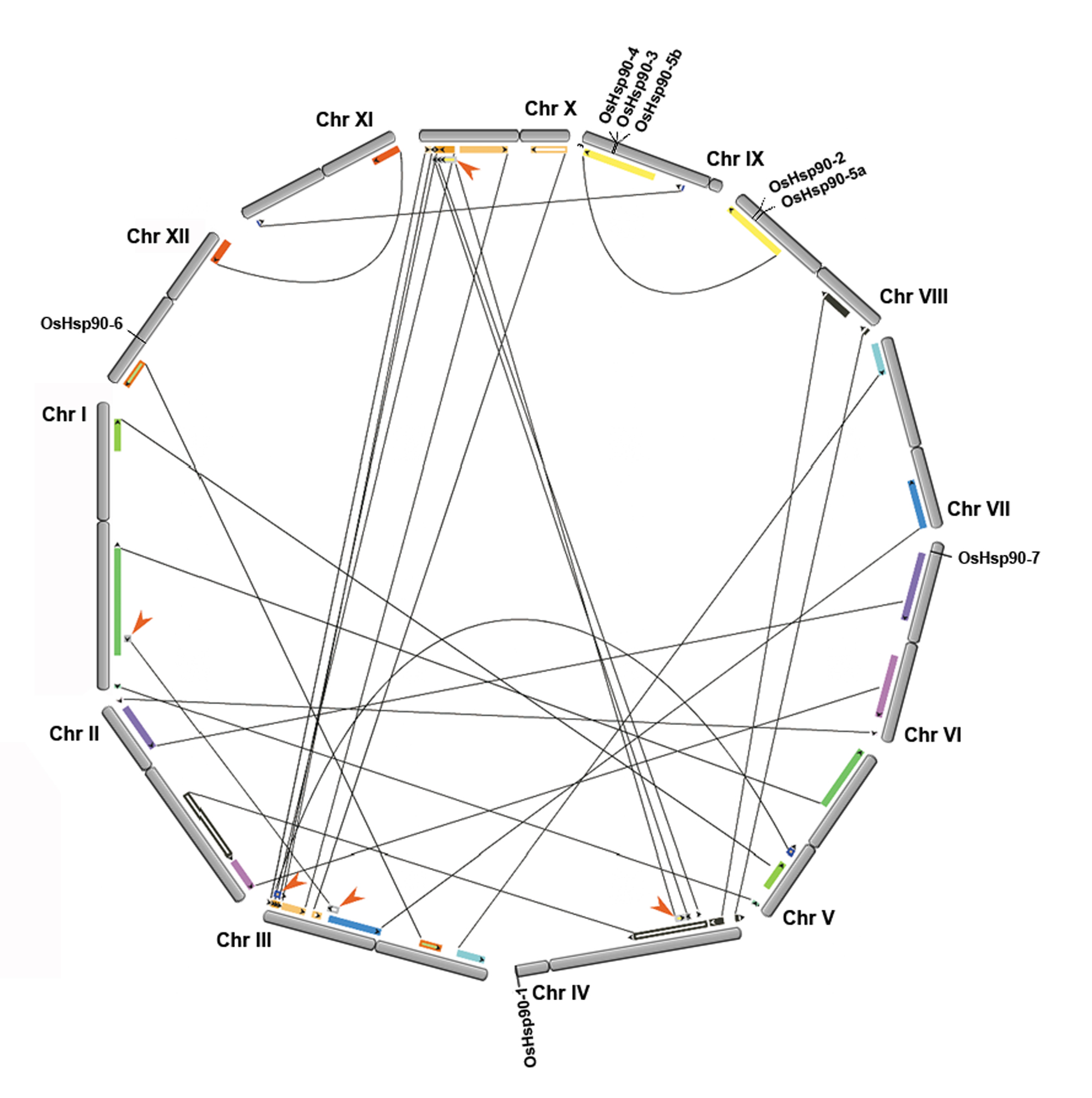

Supplement: Additional file 9 — Chromosomal locations of rice Hsp90 genes. The lines join the segmental duplicated homologous blocks that are indicated using the same colors. [file 1471-2164-14-532-S9.jpg]

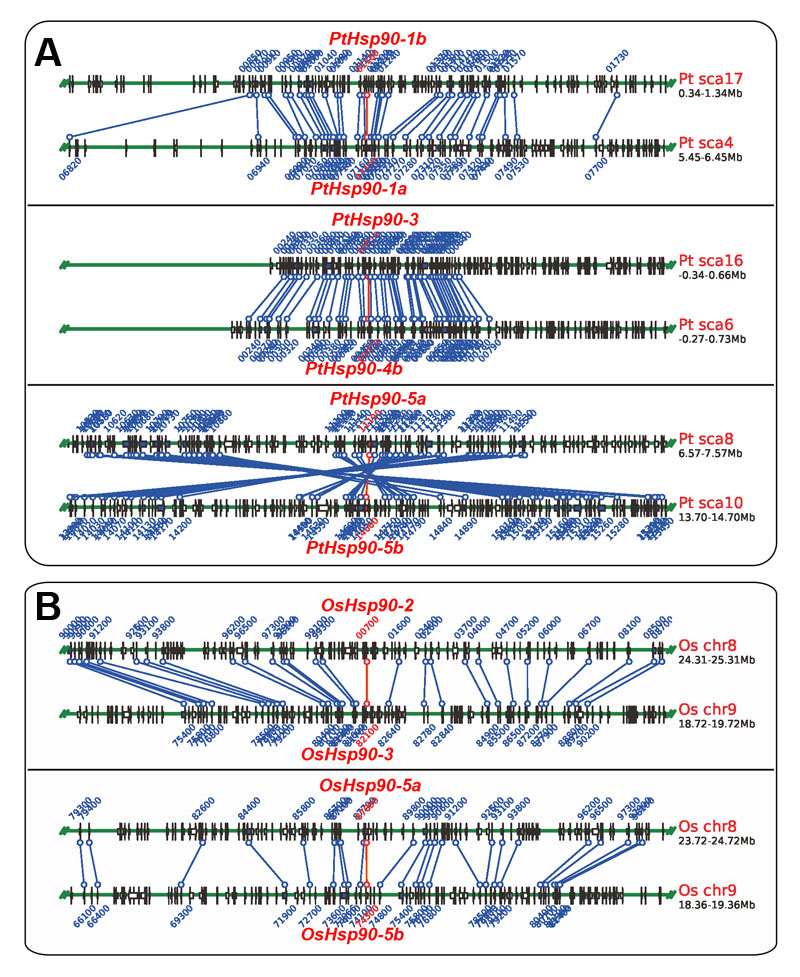

Supplement: Additional file 10 — Gene duplication relationships in the Hsp90 gene family in Populus trichocarpa and Oryza sativa. Paralogous gene pairs generated by gene duplication within the Hsp90 family of P. trichocarpa (A) and O. sativa (B) were analyzed using the Plant Genome Duplication Database (http://chibba.agtec.uga.edu/duplication/). Each query gene displays only ±500 kb regions. Gene lines connect gene pairs. Blue lines represent the other anchor gene pairs in the region, and the red line represents the query locus. [file 1471-2164-14-532-S10.jpg]

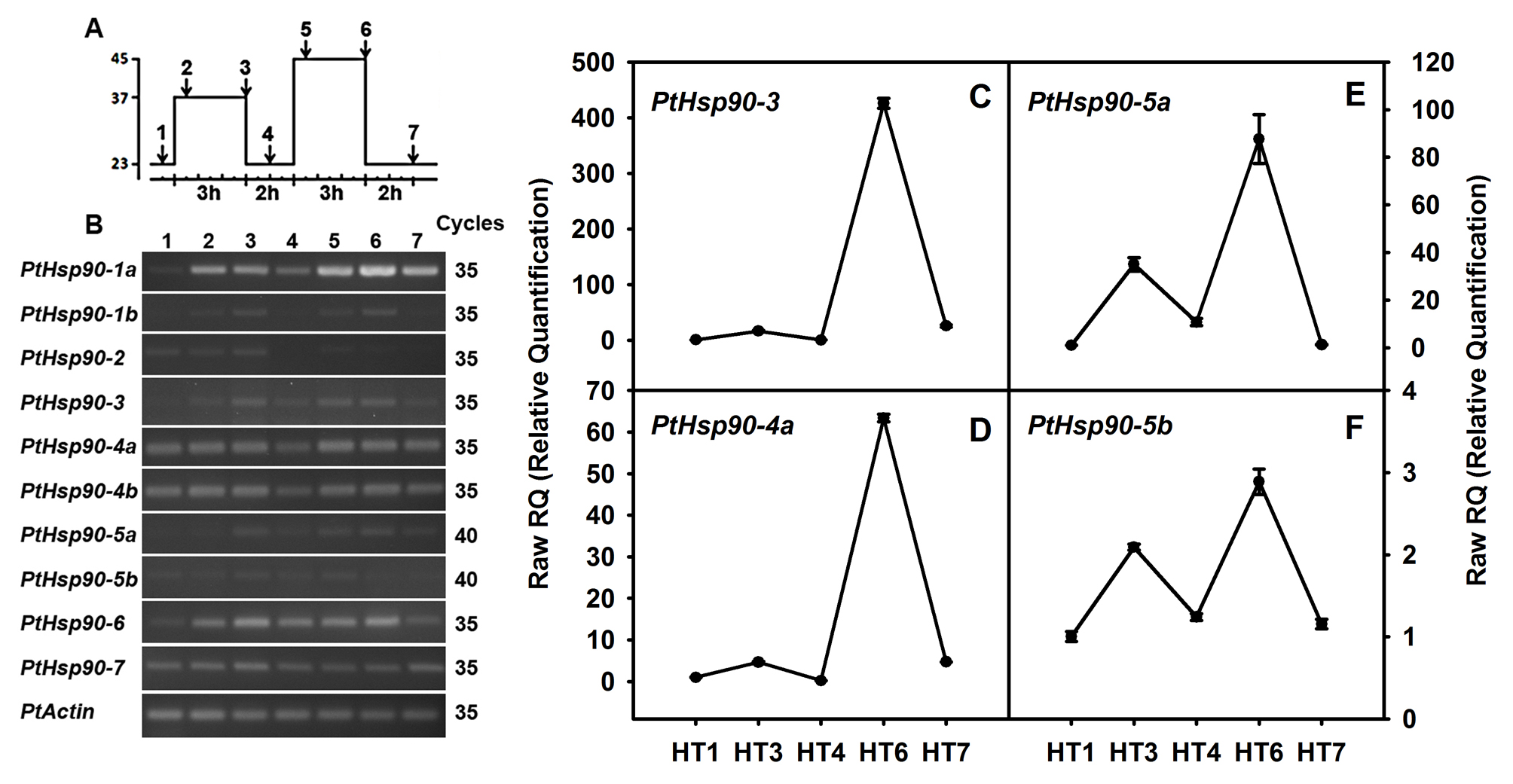

Supplement: Additional file 12 — Expression analysis of selected PtHsp90 genes under heat stress. A. Conditions of heat stress. Seedlings were heated to 37°C for 3 h (pretreatment), returned to 23°C for 2 h, heated to 45°C for 3 h (treatment), and then allowed to recover for 2 h. 1, control; 2, 30 min after pretreatment at 37°C; 3, 2 h after pretreatment at 37°C; 4, 1 h after recovery at 23°C; 5, 30 min after treatment at 45°C; 6, 2 h after treatment at 45°C; 7, 2 h after recovery at 23°C. B. Analysis of expression profiles of PtHsp90s in response to heat stress in Populus leaves by semi-quantitative RT-PCR. The constitutively expressed PtActin was used as an internal control. Three independent experiments were performed under identical conditions. C-F. The relative mRNA abundance of four selected PtHsp90 genes was normalized with respect to reference gene PtActin under heat stress using qRT-PCR. Three biological replicates each with four technique replicates were performed and bars represent standard deviations (SD) of the replicates. [file 1471-2164-14-532-S12.jpg]
